# Bimanual Use of Articulating Laparoscopic Instruments (ArtiSential) in Pediatric Surgery: A Report of Three Cases

**DOI:** 10.70352/scrj.cr.25-0809

**Published:** 2026-03-04

**Authors:** Yushi Kaisyakuji, Kahona Ono, Masateru Nakashima, Terumasa Matsuzaki, Katsuhiro Ogawa, Teijiro Hirashita, Tomonori Akagi, Shigeo Ninomiya, Yuichi Endo, Tomotaka Shibata, Hidefumi Shiroshita, Masafumi Inomata

**Affiliations:** 1 Department of Gastroenterological and Pediatric Surgery, Oita University Faculty of Medicine, Yuhu, Oita, Japan; 2 Department of Advanced Trauma, Emergency and Critical Care Center, Oita University Faculty of Medicine, Yuhu, Oita, Japan; 3 Department of Advanced Medical Personnel Nurturing, Oita University Faculty of Medicine, Yuhu, Oita, Japan

**Keywords:** pediatric surgery, ArtiSential, articulating instruments

## Abstract

**INTRODUCTION:**

Articulating laparoscopic instruments such as ArtiSential have been developed to overcome the limited range of motion associated with conventional straight instruments, particularly in narrow operative fields. While robot-assisted surgery provides highly precise manipulation, its introduction in pediatric surgery remains limited due to high cost and restricted insurance coverage. We introduced bimanual use of ArtiSential in pediatric minimally invasive surgery as a potential alternative to robotic assistance and evaluated its feasibility and technical advantages.

**CASE PRESENTATION:**

We performed laparoscopic procedures using bimanual use of ArtiSential instruments in 3 pediatric patients: 2 single-incision laparoscopic appendectomies for chronic appendicitis (10- and 15-year-old patients) and 1 laparoscopic resection of an esophageal bronchogenic cyst with Dor fundoplication (11-year-old patient). In all cases, the procedures were safely completed without intraoperative complications, and blood loss was minimal. The articulating function allowed stable triangulation and fine manipulation even in restricted spaces such as the umbilical single-port approach and the esophagogastric junction. All patients were discharged between postoperative days 3 and 4 with uneventful recovery.

**CONCLUSIONS:**

Bimanual use of ArtiSential instruments enabled precise dissection and suturing in narrow pediatric operative fields, suggesting that this approach may serve as a practical, lower-cost alternative to robotic surgery where robotic systems are unavailable or not indicated. Further accumulation of cases is required to assess the learning curve, indications, and long-term outcomes.

## Abbreviation

SILA
: single-incision laparoscopic appendectomy

## INTRODUCTION

Robot-assisted surgery has rapidly expanded in recent years, offering precise manipulation and reduced invasiveness. However, its introduction requires high installation and maintenance costs, and its insurance coverage in pediatric surgery remains limited in Japan. As a result, the availability of robotic platforms in pediatric cases is still far behind that in adult surgery. Although the use of robot-assisted surgery in pediatric surgery has gradually increased since its approval in 2019,^1)^ no nationwide statistics precisely report the annual volume. However, based on available institutional data,^1)^ it is estimated that approximately 60 pediatric cases are performed annually across roughly 10 specialized centers.

**Fig. 1:**
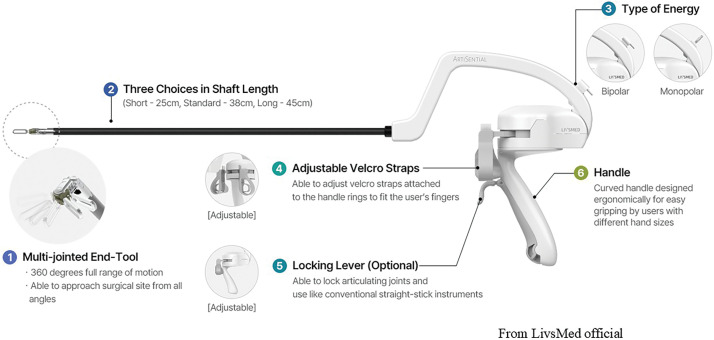
Exterior of ArtiSential (LivsMed, Seongnam, Korea). ArtiSential is a wristed, multi-degree-of-freedom laparoscopic instrument that offers robotic-like articulation with full 360° tip control while preserving the advantages of standard laparoscopy.

ArtiSential (LivsMed, Seongnam, Korea) is a newly developed articulating laparoscopic instrument equipped with a dual-joint mechanism at the distal end, allowing increased freedom of movement compared with conventional straight instruments (**Fig. 1**). Its mechanical design enables manipulation similar to that of robotic wristed instruments while maintaining the cost advantages of standard laparoscopy.^2)^ Specifically, ArtiSential provides a total of 7 degrees of freedom through its dual-joint distal mechanism, which is comparable to robotic wristed instruments such as the da Vinci EndoWrist (Intuitive Surgical, Sunnyvale, CA, USA), whereas conventional laparoscopic forceps generally offer only 4 to 5 degrees of freedom. These characteristics are expected to be particularly useful in pediatric cases, where the operative field is small, as well as in single-incision, mediastinal, or deep pelvic surgery.^3)^ However, bimanual use of ArtiSential requires simultaneous control of shaft motion (reverse-phase) and end-effector joint motion (in-phase), resulting in 4 degrees of movement that demand dedicated learning and hand-eye coordination. Despite this, once mastered, the technique has the potential to provide robotic-level maneuverability without the need for a robotic platform.

We describe 3 pediatric cases in which this technique was applied and evaluate its feasibility and surgical advantages.

## CASE PRESENTATION

### Case 1

**Fig. 2:**
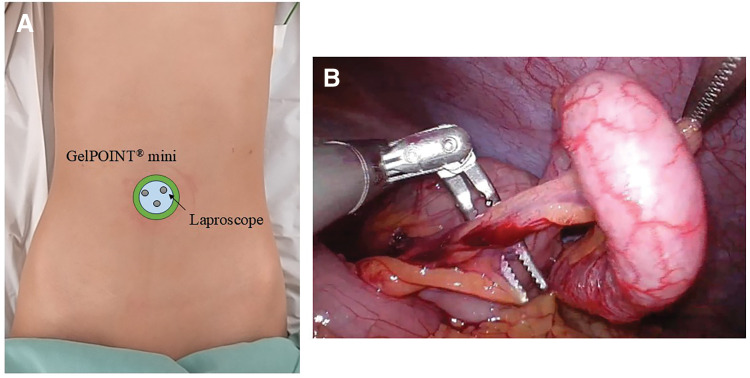
Case 1. **(A)** Port placement for Case 1. **(B)** Intraoperative findings of Case 1, in which the appendiceal vessels were dissected at a right angle without shaft manipulation or interference with the laparoscope.

A 10-year-old girl (height, 142 cm; weight, 31.4 kg) presented with recurrent right lower abdominal pain and was diagnosed with chronic appendicitis based on clinical findings and imaging. An SILA was performed through a 20-mm umbilical incision using a GelPOINT (Applied Medical, Rancho Santa Margarita, CA, USA) Mini port, a 5-mm rigid laparoscope, and two 5-mm ArtiSential instruments (**Fig. 2A** and **2B**). The appendiceal artery and mesoappendix were divided using LigaSure (Medtronic, Minneapolis, MN, USA) at the root, and the appendiceal stump was double-ligated with an ENDOLOOP Ligature (Ethicon, Somerville, NJ, USA) before transection. The procedure was completed entirely laparoscopically without exteriorization of the cecum. The total operative time was 78 minutes, and intraoperative bleeding was minimal (**Supplementary Video 1**, Case 1). The postoperative course was uneventful, and the patient was discharged on POD 3.

### Case 2

**Fig. 3:**
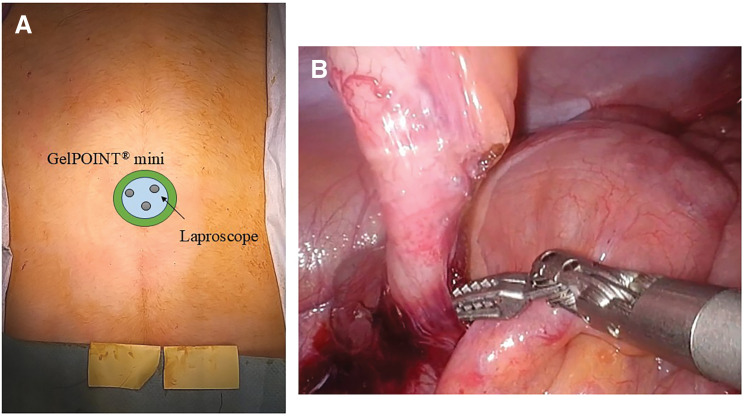
Case 2. **(A)** Port placement for Case 2. **(B)** Intraoperative findings of Case 2.

A 15-year-old boy (height, 166 cm; weight, 49.2 kg) with chronic appendicitis underwent the same SILA procedure using the identical port setting and bimanual ArtiSential manipulation (**Fig. 3A** and **3B**). A LigaSure vessel sealing device was used for tissue sealing. The procedure was completed entirely laparoscopically without exteriorization of the cecum. The total operative time was 67 minutes, and intraoperative bleeding was minimal (**Supplementary Video 1**, Case 2). He was discharged on POD 3 without postoperative complications.

### Case 3

**Fig. 4:**
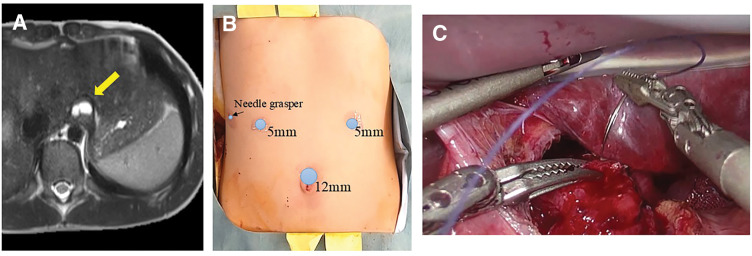
Case 3. **(A)** Preoperative MRI, demonstrating a 15-mm cystic lesion on the anterior wall of the abdominal esophagus. Based on its location and homogeneous fluid content, a bronchogenic cyst was diagnosed preoperatively. **(B)** Port placement for Case 3. **(C)** Intraoperative findings. After cyst excision, the defect in the muscular layer was closed in the short-axis direction, allowing tangential suturing relative to the laparoscopic camera.

An 11-year-old boy (height, 154 cm; weight, 35.6 kg) was diagnosed with an esophageal bronchogenic cyst located anterior to the abdominal esophagus (**Fig. 4A**). Laparoscopic cyst resection followed by Dor fundoplication was performed. A 15-mm umbilical incision was used for a 12-mm trocar, and two 5-mm working ports were placed in the upper abdomen. A 3-mm needle grasper was inserted through the right abdominal wall as an auxiliary instrument (**Fig. 4B**), and the left lateral segment of the liver was retracted externally with a silicone disk and nylon suture. A LigaSure vessel sealing device was used for tissue sealing.

After circumferential mobilization of the abdominal esophagus, the cyst was excised together with the adherent seromuscular layer. The resulting defect was closed with 3-0 Vicryl (Ethicon) interrupted sutures in the short-axis direction (**Fig. 4C**), followed by Dor fundoplication for reinforcement (**Supplementary Video 1**, Case 3). Operative time was 347 minutes, and the patient was discharged on POD 4 without complications.

### Technical note on instrument handling

In all 3 cases, both ArtiSential instruments were operated bimanually by the same surgeon. Because the 5-mm ArtiSential lineup did not include a sealing device or forceps-type electrosurgical instrument at the time of surgery, LigaSure was used as the energy device. The articulating mechanism allowed smooth triangulation and stable suturing, even in confined spaces such as the esophagogastric junction and single-port SILA setting. No instrument collision with the laparoscope occurred, and the need for excessive shaft manipulation was reduced compared with conventional straight instruments.

All procedures were completed safely without conversion to additional port or open surgery (**Table 1**). Estimated blood loss was minimal in all cases, and no intraoperative or postoperative complications occurred. The articulating function enabled active control of needle angle and tissue traction, improving ergonomic stability in narrow operative fields. In abdominal esophageal surgery, tangential suturing could be performed precisely without interference from the laparoscope or other instruments. In conventional laparoscopic forceps, the needle driving direction is not aligned with the instrument shaft axis. By contrast, the articulating joint of ArtiSential allows alignment of the motion axis with the needle driving direction, facilitating more precise and controlled needle placement. In appendectomy, the articulating mechanism allowed multidirectional dissection of the appendiceal base within a single visual field while minimizing shaft movement. The procedure could be completed entirely laparoscopically without the need for exteriorization of the cecum, allowing a stable single-incision approach regardless of patient body habitus or age.

**Table 1:** Clinical characteristics of the three cases

Case	Age	Height (cm)	Body weight (kg)	Operative time (min)	Pneumoperitoneum time (min)	Postoperative complications	Time to oral intake (POD)	Length of hospital stay (days)	Type of ArtiSential used
1	10	142	31.4	78	34	None	1	4	5-mm Fenestrated Forceps
									5-mm Maryland Dissector
2	15	166	49.2	67	42	None	1	4	5-mm Fenestrated Forceps
									5-mm Maryland Dissector
3	11	154	35.6	347	295	None	2	5	5-mm Fenestrated Forceps
									5-mm Maryland Dissector
									5-mm Needle Holder

## DISCUSSION

These cases suggest that bimanual use of the ArtiSential articulating laparoscopic instrument may offer clinical utility in pediatric minimally invasive surgery. A comprehensive search of the literature identified a total of eight reports describing the clinical use of ArtiSential published in English-language journals worldwide (**Table 2**). Of these, 7 reports originated from South Korea, and 1 from Germany. Among these studies, only one clearly specified whether the instrument was used unilaterally or bimanually, and that report described unilateral use only. With regard to surgical fields, 1 report involved gastric surgery, 3 involved colorectal surgery, 1 involved pancreatic surgery, 1 involved renal surgery, and 2 involved gynecologic surgery. To the best of our knowledge, no previous studies have reported clinical applications limited to bimanual use of ArtiSential, nor have there been any reports describing its use in the field of pediatric surgery. In this respect, the present report represents the first description of pediatric laparoscopic procedures performed using bimanual application of ArtiSential.

**Table 2:** Previously published English reports on the clinical use of ArtiSential

No.	Author	Country	Year	Field	Single or bimanual
1	Darwich et al.^3)^	Germany	2022	Colorectal surgery	Not stated
2	Kang et al.^9)^	Korea	2023	Gastric surgery	Single manual
3	Pyo et al.^12)^	Korea	2024	Colorectal surgery	Not stated
4	Kim et al.^13)^	Korea	2024	Renal surgery	Not stated
5	Kang et al.^14)^	Korea	2024	Gynecologic oncologic surgery	Not stated
6	Won et al.^15)^	Korea	2024	Gynecologic surgery	Not stated
7	Jeong and Kang^16)^	Korea	2024	Pancreatic surgery	Not stated
8	Ahn et al.^17)^	Korea	2025	Colorectal surgery	Not stated
Ours	Kaisyakuji et al.	Japan	2025	Pediatric surgery	Bimanual

All 3 procedures were performed in narrow operative fields—2 single-incision appendectomies and 1 esophagogastric procedure—and were completed successfully without complications. A key feature of ArtiSential is its dual-joint structure, which provides enhanced maneuverability near the instrument tip; this characteristic has also been demonstrated in previous evaluations of multi-degree-of-freedom articulating laparoscopic instruments.^2,3)^ This structural design enables multidirectional manipulation resembling that of robotic-assisted surgery. In adult gastrointestinal, colorectal, and hepatobiliary surgery, articulating laparoscopic instruments including ArtiSential have been reported to be safely applicable with favorable perioperative outcomes,^4,5)^ and comparative studies in colorectal surgery have demonstrated perioperative results comparable to those of robotic-assisted surgery.^6)^

Pediatric surgery presents distinct technical challenges compared with adult surgery, including limited intra-abdominal working space, narrow trocar spacing, and the need to avoid excessive organ compression. Under such conditions, the present report suggests that bimanual use of ArtiSential may expand the applicability of single-incision and deep-field laparoscopic procedures without the use of a robotic platform. Previous pediatric reports have been limited to single-instrument use, and the present cases add complementary evidence to this emerging field. In addition, the reduced requirement for exaggerated shaft movements contributed to stable visualization and minimized interference with the laparoscope. All 3 patients in the present series were of school age or older, and the available operative workspace was adequately maintained compared with that in younger pediatric patients. In theory, given that the distance from the articulating joint to the instrument tip is 3.0 cm, surgery including suturing can be performed within a quarter-spherical workspace with a radius of about 4.0 cm. In our cases described herein, once a stable operative field had been established, lateral shaft movements were rarely required, and the procedures were completed predominantly through tip manipulation distal to the articulating joint mechanism (**Supplementary Video 1**). Based on these findings, we consider that this approach is also sufficiently applicable to surgical cases in younger pediatric patients, including infants. In recent years, robotic surgery has been reported to be safely performed in infants and neonates.^7)^ In robotic systems, external arm collision is considered to be one of the factors that may limit their applicability in patients with small body size; however, the feasibility of robotic surgery has been demonstrated even in patients weighing less than 5 kg. Based on this evidence, articulating laparoscopic instruments such as ArtiSential, which provide a comparable joint mechanism without external arm interference, may theoretically be applicable to surgical cases in infants and neonates. Taken together, bimanual use of ArtiSential may contribute to the expansion of indications for further minimally invasive approaches, such as single-incision surgery and reduced-port surgery, in pediatric surgery, while also enabling precise manipulation comparable to robotic surgery even in confined operative fields including infants and neonates.

Economic considerations are also important. Rising healthcare costs represent a common challenge worldwide, including in Japan, where robotic surgery requires substantial financial investment for both introduction and maintenance. Kajiwara et al. reported that the initial investment for robotic surgery was approximately 250 million yen, with annual maintenance costs of approximately 10 million yen and per-case costs of approximately 700000 yen, including consumables.^6)^ These estimates do not include expenses related to operating room renovation, acquisition and maintenance of peripheral equipment, or training of medical staff, suggesting that the actual financial burden may be even greater. In addition, the period from introduction to the first clinical case has been reported to require approximately 1–1.5 years.^6)^ By contrast, ArtiSential does not require facility renovation in institutions already performing laparoscopic surgery, and the time required for introduction is largely dependent on surgeon training. At our institution, the per-case cost associated with ArtiSential use was less than 100000 yen, and the interval from the decision to introduce the device to the first clinical application in pediatric surgery was 26 days. The cost-effectiveness of articulating laparoscopic instruments has also been demonstrated in previous reports.^8)^

Thus, by enabling precise manipulation comparable to robotic surgery while allowing low-cost and rapid introduction, articulating laparoscopic instruments may represent a useful option for institutions, regions, or surgical fields where robotic surgery has not been introduced or is difficult to implement because of economic or infrastructural constraints. A recent report has likewise positioned articulating laparoscopic systems as a bridge between conventional laparoscopy and robotic surgery.^9)^ Accordingly, these findings suggest that ArtiSential may reduce barriers to advanced minimally invasive surgery and has the potential to contribute to mitigating regional and institutional disparities in surgical care.

Nevertheless, several limitations should be acknowledged. First, bimanual manipulation involves a learning curve, as it requires simultaneous control of multiple axes, including reverse-phase shaft movement and in-phase wrist articulation. In the present report, approximately 2 hours of dry-laboratory grasping training and 4 hours of intracorporeal suturing practice were required before clinical application. Previous studies evaluating training for unilateral use have shown that a 30-minute training session can reduce task completion time and narrow performance differences among experts, intermediates, and novices, and that several hours of practice (up to 8 hours) may result in shorter task completion times than those achieved with conventional laparoscopic instruments.^10)^ On the other hand, in a prospective study using a dry box conducted by Murakami et al., 42.5 ± 15.7 minutes were required to complete 3D peg transfer and safe vertical suturing with the ArtiSential instrument.^11)^ These findings are consistent with the experience described in this report. However, for bimanual use, further establishment and standardization of effective training methods will be necessary. Second, this report includes only 3 cases with short-term outcomes. Further accumulation of cases is required to evaluate learning curves, reproducibility, and surgical outcomes more comprehensively. Finally, ArtiSential is a relatively new device, and a number of advantages and limitations have been reported in the literature to date. These are summarized in **Table 3**.

**Table 3:** Advantage and disadvantage of ArtiSential

Advantage	Disadvantage
• Robotic-like articulating freedom^1)^ • Comparable performance to robotic surgery^1)^ • Haptic feedback^1)^ • Shorter operative time than robotic surgery^4,5)^ • Articulation achievable without high robotic cost^8)^ • Learning curve reaches a plateau after several hours^10)^ • Lower open conversion rate (vs. conventional)^12,14)^	• Greater wrist discomfort than robotic surgery^1)^ • Heavy instrument weight^1,14)^ • Slight trend toward longer operative time (not significant)^12)^ • Lack of dedicated scissors and clip appliers for this instrument^13)^ • Instrument insertion/removal is difficult^14)^

Despite these limitations, the bimanual use of ArtiSential supports the concept that articulating laparoscopic instruments may serve as a practical alternative to robotic surgery in disease entities and surgical fields where robotic systems are unavailable, economically challenging, or not covered by insurance.

## CONCLUSIONS

Bimanual use of the ArtiSential articulating instruments contributes to the expansion of single-incision surgery and the advancement of reduced-port surgery, thereby promoting further minimization of surgical invasiveness. This technique enables precise maneuvers, including tangential suturing in confined operative fields such as abdominal esophageal surgery, without interference from the laparoscope or other instruments. Moreover, it allows the realization of surgical performance comparable to robotic-assisted surgery at substantially lower cost and with rapid implementation.

In the present report, bimanual application of ArtiSential enabled stable visualization and precise manipulation and suturing even in narrow pediatric operative fields, including single-incision and esophagogastric procedures. This technique may represent a cost-effective alternative to robotic surgery in pediatric cases. Further accumulation of clinical experience is required to clarify appropriate indications, learning curves, and long-term outcomes.

## SUPPLEMENTARY MATERIALS

Supplementary Video 1 Representative intraoperative findings in Cases 1–3. This video presents representative intraoperative scenes from Cases 1–3. In Cases 1 and 2, bimanual manipulation with ArtiSential during laparoscopic appendectomy enabled multidirectional dissection within a single stable view with minimal interference with the laparoscopic camera. In Case 3, closure of the muscular defect after cyst excision is demonstrated, highlighting precise tangential suturing enabled by articulating instruments in a narrow pediatric esophagogastric space.

## DECLARATIONS

### Use of artificial intelligence (AI) tools

The author used ChatGPT 5.1 (OpenAI, San Francisco, CA, USA) to assist with English translation and language editing of this manuscript. All scientific content, clinical interpretations, and final editorial decisions were made solely by the author.
